# NATRIURETIC PEPTIDE SYSTEM AND CARDIOVASCULAR DISEASE

**Published:** 2010

**Authors:** Cacciapuoti Federico

**Affiliations:** * *Associate Professor of Internal Medicine, Second University of Naples, Italy*

**Keywords:** Natriuretic Peptides, heart failure, left ventricular remodeling, vasoconstriction, Nesiritide, CNP-like drugs, Endopeptidase, Vasopeptidase

## Abstract

The mammalian Natriuretic Peptide (NP) system consists of neuro-hormones, such as atrial natriuretic peptide (ANP), brain natriuretic peptide (BNP), c-type natriuretic peptide (CNP), and the N-Terminal fragment of BNP (NT-pro-BNP). In response to some cardiovascular derangement the heart (acting as an endocrine organ), brain and other structures secretes natriuretic peptides in an attempt to restore normal circulatory conditions. Their actions are modulated through membrane-bound guanylyl cyclased (GC) receptors. They induce diuresis, natriuresis and vasodilation in the presence of congestive heart failure. These neuro-hormones also play a role in the suppression of neointimal formation after vascular injury. In addition, they act as antifibrotic and antihypertrophic agents preventing cardiac remodeling after myocardial infarction. Further, NP have diagnostic and prognostic role in heart failure, vasoconstriction, left ventricular late remodeling after MI and others. At present, some drugs such as Nesiritide, NEP inhibitors and vasopeptidase inhibitors were synthetized from NP, to antagonize these cardiovascular derengements. In future, it will be possibile to elaborate some drugs similar to petidase inhibitors and some CNP-like drugs able to reduce many symptoms of cardiovascular derangements without significant side effects.

## Introduction

**Fig 1:**
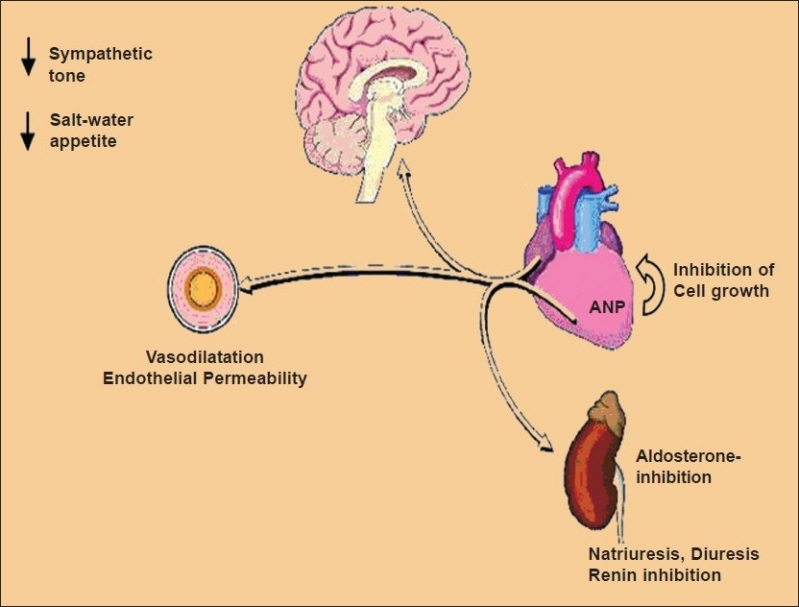
The leading actions of Natriuretic Peptides in to reducing the water and salt retention (oedema) and vasoconstriction

Natriuretic Peptide (NP) System is composed of neurohormones synthesized by the heart, brain and other organs^1^. Numerous cardiovascular diseases, such as chronic heart failure, systemic hypertension, coronary disease, endothelial dysfunction and others are responsible for their raised secretion, in an attempt to normalize the state of cardiovascular health. Their actions are modulated through membrane-bound receptors, two of which are guanylyl cyclase (GC)-coupled receptors (GC-A and CG-B). These are linked to the cyclic guanosine monophosphate (cGMP)-dependent signaling cascade^2^. The neuro-hormones are involved in the long-term regulation of sodium and water balance, blood volume and arterial pressure. In addition, NP directly dilate veins and decrease central venous pressure, reducing cardiac output. They also increase glomerular filtation rate (GFR) and filtration fraction, acting at the renal level. As a consequence, NP favors natriuresis and diuresis, decreasing edema. Another renal action is the reduction of renin release, by decreasing circulating levels of Angiotensin II and Aldosterone. That further increases natriuresis and diuresis. These actions on SRAA contribute to systemic vasodilation and decrease systemic vascular resistance (Fig.1).

## Natriuretic Peptide System

At present, the mammalian natriuretic peptide system consists of some substances: Atrial Natriuretic Peptide (ANP); Brain Natriuretic Peptide (BNP); C-type Natriuretic Peptide (CNP)^2^–^4^.N Terminal fragment of pro BNP (NT-pro-BNP) must be also considered. This last derives from the proteolysis of proform BNP.

### Atrial Natriuretic Peptide (ANP)

ANP is the first discovered member of this family. The major site of synthesis in normal heart is the atrium, and secretion is stimulated by stretch. In normal adult heart, ventricular tissue produces only minor amounts of ANP, but major amounts are found in ventricles of fetuses and in patients with LV hypertrophy. ANP is a 28-amino acid peptide stored and released by atrial myocytes in response to atrial distension and stretch. In addition, it also increases angiotensin II and/or endothelin rise-concentrations as a consequence of sympathetic stimulation.

Elevated levels of ANP generally are found in hypervolemic states inducing a condition of congestive heart failure. It is present in patients with heart failure and chronic atrial fibrillation (AF). In this connection, ANP levels were higher among those with AF of longer duration than in decompensated patients with AF of shorter length^5^. In addition, ANP provides prognostic significance in patients with chronic heart failure. With regard to this, Gottlieb et al. found that the neurohormone serum-levels are connected with the numbers of premature ventricular depolari-zations occurring in decompensated heart failure, plasma levels renin or norepinephrine concentrations^6^. Recently, it has been demons-trated that the improvement in hemodynamic function in patients with chronic heart failure by Ramipril is associated with a decrease of ANP levels^7^.

### Brain Natriuretic Peptide (BNP)

BNP was primarily discovered in porcine brain, but the highest concentration of peptide is found in the heart. It is a 32 amino-acid peptide, synthesized within the ventricles in response to myocyte stretch and/or pressure overload8. It is released both as an active hormone and an inactive N-terminal fragment (NT pro-BNP). Recently, Elnomany and Abdelhameed found that BNP levels are elevated in patients with symptomatic LV dysfunction. The reduction of Mitral Annulus Posterior Excursion (MAPSE), as the index of left ventricular dysfunction, appears strongly correlated with plasma BNP levels. This correlation provides a simple, accurate and reproducible tool for early diagnosis of LV dysfunction^9^.

In addition, BNP levels are higher in patients with dyspnea (shortness of breath) due to heart failure than in patients with dyspnea induced by other causes. Consequently, BNP measurement in the emergency room helps in to discriminate two types of dyspnea. Besides, BNP appears to be a useful marker of cardiovascular risk, even in persons without clinical evidence of cardiovascular disease. Finally, BNP levels were employed to predict the risk of heart failure atrial fibrillation and stroke or transient ischemic attack. Conclusively, the hemodynamic effects of BNP are largely similar to those of ANP.

### C-type Natriuretic Peptide (CNP)

C-type Natriuretic Peptide was originally isolated from porcine brain extracts but, the major site of synthesis are the vessel walls. The peptide plays a role in the suppression of neo-intima formation after injury^10^. In addition, it has a more potent anti-hypertrophic, antifibrotic and anti-proliferative effects and hence, useful in prevent late cardiac remodeling after MI^11^. The beneficial effects of CNP on the heart after MI includes an attenuation of cardiac fibrosis, hypertrophy and LV enlargement. Therefore, CNP acts both on endothelial and on ventricular levels. Its beneficial effects seems to be due to the direct inhibition of DNA and collagen synthesis of cardiac fibroblasts^12^. Although the precise inhibiting-mechanisms of CNP remains unknown, previous studies suggested that CNP inhibits hypertrophy of cardiac myocytes directly by activating cGMP-dependent mechanism and indirectly by reducing endothelin-1 secretion from non-myocytes^13^.

Numerous compounds limiting ventricular remodeling after MI heve been reported. These include angiotensin-converting enzyme inhibitors, angiotensin II type 1 receptor blockers, aldosterone antagonists, and matrix metalloproteinase inhibitors. Although almost all these agents have been given orally contrary to CNP, this last has the advantage concerning short period of treatment and fewer side effects.

### N Terminal fragment of -pro BNP (NT-pro-BNP NT)

N-terminal fragment of Brain Natriuretic Peptide (NT pro-BNP) derives from the proteolysis of pro-BNP (composed of 108 amino-acids). It consists of 76 amino-acids and recently caused great interest for its possible role in monitoring cardiac insufficiency^14^and in the stratification of acute coronary syndromes (ACS)^15^. Its effects on diuresis and natriuresis (in patients with congestive heart failure) represent a “compensatory” mechanism to the stress of myocytes evolving in ventricular dysfunction. In unstable angina NT pro-BNP represents an efficacious marker of the damage caused by cardiac ischemia. The severity of the coronary disease is evidenced by an increase of NT -proBNP levels. In addition, in acute coronary syndromes (ACS), NT-proBNP has a immunomodulant role^16^and provides important prognostic information in patients evolving to heart failure^17^.

## Diagnostic and prognostic value of NP

The NP serum levels are important not only as indicators of numerous cardiovascular derangements, but also as markers of their severity. ANP are found elevated in patients suffering from MI and having congestive heart failure^18^. In addition, it was found that intravenous administration of ANP in heart failure notably improved cardiac nerve sympathetic activity and left ventricular remodeling^19^. Finally, in patients with Acute Coronary Syndromes (ACS), BNP measurement provides predictive information in risk stratification in the absence of S-Televation^20^. Besides ACS, BNP and NT-proBNP have prognostic significance in acute pulmonary embolism.

This diagnostic value was recently confirmed by Coutance et al. Although elevated BNP levels have a high sensitivity to detect patients at risk of death, the specificity of this neuro-hormone is low^21^. A multivariate analysis between mortality and BNP levels was recently performed by Nunez and coworkers, demonstrating a positive linear correlation between the risk of death and BNP^22^. With regard to prognostic value of N-Terminal-proBNP in chronic heart failure, Va-HeFT Trial demostrated its positivity in high degree advanced cardiac failure^23^. Finally, BNP concentration appears significantly raised in patients with dilated cardiomyopathies and cardiovascular disease in NYHA classes III or IV but it did not predict mortality or the necessity of heart transplant^24^.

## New NP-derived drugs

The beneficial effects of NP on cardiovascular disease stimulated the development of new compounds able to prolong and enhance these positive effects. Of these:

***Nesiritide*** is a recombinant form of human BNP, approved for use in the acute treatment of congestive heart failure caused by systolic dysfunction^25^. It increases intracellular cyclic-GMP in vascular smooth muscle cells, leading to smooth muscle relaxation, pre-load and after-load reduction, and increased cardiac index in patients with congestive heart failure. The drug has been evaluated in clinical trials involving more than 700 patients. In these trials, Nesiritide produced a prompt fall in systemic vascular resistance and pulmonary capillary “wedge” pressure, associated with rapid improvement in decompensated heart failure. Therefore, the compound seems to represent an attractive choice for decompensated heart failure therapy^26^. But, two meta-analyses demonstrated that Nesiritide might lead to worsening renal function^27^and increased mortality^28^. In addition, treatment with Nesiritide was associated with 74% increased risk of death within 30 days. These conflicting results perhaps are consequent to short term follow-up (30 days), noninotrope-based control therapies, and closed-label trial design. In spite of these considerations, the US FDA approved Nesiritide for the teatment of acutely decompensated heart failure (ADHF).

The agent is indicated for intravenous treatment and has advantageous, pluripotent properties in ADHF including hemodynamic, neurohormonal, lusitropic, and reverse remodeling effects. In addition, it was satisfactorily compared with other vasodilating agents and did not promotes arrhythmogenesis^29^.

Subsequently, a number of other drugs deriving from CNP have been prepared^30^,^31^But, contrary to other approaches (such as angiotensin-converting enzyme inhibitors, adrenergic blockers, aldosterone antagonists or matrix metallopreteinase inhibitors), CNP-like drugs have some advantages concerning short treatment period and fewer side effects^32^. Neutral endopeptidase (NEP) are circulating enzymes able to degrade NP. On the contrary, its inhibition increases circulating levels of NP and potentiates their negative effects. Thus, the NEP inhibition avoids the actions of NP.

More recently, a new class of drugs similar to NEP inhibitors have been shown to be efficacious in animal models with heart failure. Treatment with NEP-inhibitor, Candoxantril, increases urinary sodium and significantly elevates filtration fraction with no significant effect on glomerular filtration rate, renal plasma flow or lithium clearance. A reduction in aldosterone concentration is also evident in these patients^33^. It acts by inhibiting NEP and ACE and is employed for the treatment of Systemic Hypertension in patients with CHF. In addition, the drug has a tissue protective effects on fibroblasts growth (antiremodeling effect). These drugs, were also combined with ACE-inhibitors in a single molecule. This strategy is known such as vasopeptidase inhibition. It offers the prospect of combining the benefits of increased NP levels with those of ACE-inhibition. These compounds simultaneously inhibit the activity of ACE and NEP, representing a therapeutic advantage^34^,^35^.

There are, however, complex interactions between ACE and NEP inhibition. Both ACE and NEP metabolize the kinin peptides bradykinin and kallidin, whereas NEP converts angiotensin I to angiotensin and metabolizes Angiotensin II and endothelin. Addition of NEP inhibition to ACE inhibition potentiates the ACE-inhibitor-induced increase in kinin levels. But, the combined ACE/NEP inhibition increases the risk of angioedema and may counteract any benefit of ACE inhibition^36^.

## Conclusion and future directions

Conclusively, NP is an endogenous system able to induce most common circulatory derangements, such as water retention, vasoconstriction in response to CHF. In addition, the system causes endothelial dysfunction and left ventricular late remodeling. To avoid these negative effects, new derived drugs are recently prepared to reduce, eliminate or delay the symptoms of cardiac and vascular impairments. They act by performing some cardiovascular and renal actions such as: natriuresis; increase of glomerular filtration; systemic vasodilation; inhibition of renin release; reduction of left ventricular remodeling; reduction of venous and “wedge” pressure.

These drugs must be able to attain, extend, and stabilize neuro-hormonal, hemodynamic and clinical improvements of symptoms of some cardiovascular disease, such as chronic heart failure, myocardial infarction or systemic hypertension. In future, these drugs will become more and more important, because they act by addressing common cardiovascular symptoms through endogenous principles, which allows resolution of symptoms or retard progression of symptoms without adverse side effects.
